# The impact of passive case detection on the transmission dynamics of gambiense Human African Trypanosomiasis

**DOI:** 10.1371/journal.pntd.0006276

**Published:** 2018-04-06

**Authors:** Francesco Checchi, Sebastian Funk, Daniel Chandramohan, François Chappuis, Daniel T. Haydon

**Affiliations:** 1 Department of Infectious Disease Epidemiology, London School of Hygiene and Tropical Medicine, London, United Kingdom; 2 Centre for the Mathematical Modelling of Infectious Diseases, London School of Hygiene and Tropical Medicine, London, United Kingdom; 3 Department of Disease Control, London School of Hygiene and Tropical Medicine, London, United Kingdom; 4 Division of Tropical and Humanitarian Medicine, Geneva University Hospitals, Geneva, Switzerland; 5 Institute of Biodiversity, Animal Health and Comparative Medicine, College of Medical, Veterinary and Life Sciences, University of Glasgow, Glasgow, United Kingdom; Institut Pasteur, FRANCE

## Abstract

Gambiense Human African Trypanosomiasis (HAT), or sleeping sickness, is a vector-borne disease affecting largely rural populations in Western and Central Africa. The main method for detecting and treating cases of gambiense HAT are active screening through mobile teams and passive detection through self-referral of patients to dedicated treatment centres or hospitals. Strategies based on active case finding and treatment have drastically reduced the global incidence of the disease over recent decades. However, little is known about the coverage and transmission impact of passive case detection. We used a mathematical model to analyse data from the period between active screening sessions in hundreds of villages that were monitored as part of three HAT control projects run by Médecins Sans Frontières in Southern Sudan and Uganda in the late 1990s and early 2000s. We found heterogeneity in incidence across villages, with a small minority of villages found to have much higher transmission rates and burdens than the majority. We further found that only a minority of prevalent cases in the first, haemo-lymphatic stage of the disease were detected passively (maximum likelihood estimate <30% in all three settings), whereas around 50% of patients in the second, meningo-encephalitic were detected. We estimated that passive case detection reduced transmission in affected areas by between 30 and 50%, suggesting that there is great potential value in improving rates of passive case detection. As gambiense HAT is driven towards elimination, it will be important to establish good systems of passive screening, and estimates such as the ones here will be of value in assessing the expected impact of moving from a primarily active to a more passive screening regime.

## Author summary

Gambiense Human African Trypanosomiasis, or sleeping sickness, is transmitted by the tsetse fly and affects rural populations in Western and Central Africa. It is a deadly disease if untreated, and it is therefore important to find people in the early stages of disease so that appropriate care and medication can be provided. Because of this, much emphasis is put on mobile teams going from village to village and actively finding as many potential patients as possible. This does not reach all infected people, though, and some are only detected passively, that is they report themselves to a health provider, often in advanced stages of disease. It is not clear what proportion of cases of sleeping sickness are detected in this way, or how much onwards transmission is prevented. Here we used a mathematical model to analyse data from a sleeping sickness control programme in Uganda and South Sudan, in order to identify which proportion of people infected with the disease are identified through passive case detection. We found that only a minority of patients are identified in this way in the early stages of disease, but around half are identified if they are in the later stages. We further found that passive screening reduced transmission in affected areas by between 30 and 50%. This suggests that there is great potential value in improving the rates of passive case detection, and we recommend that more emphasis is put on tackling potential barriers that prevent people being detected.

## Introduction

Human African Trypanosomiasis (HAT, sleeping sickness) is a vector-borne disease caused by parasites of the species *Trypanosoma brucei* and transmitted by flies of the genus *Glossina*. The West African form (gambiense HAT) is a target for elimination as a public health problem by 2020, with a view to zero incidence by 2030 [1].

While active case detection has been a key strategy to bring gambiense HAT towards elimination, passive case detection is a basic element of nearly all gambiense HAT control programmes [2]. Even in programmes that are mainly focused on active screening, patients can self-refer to the fixed HAT treatment centres that must be established in order to administer the complicated treatment regimens required for HAT case management.

The coverage of passive case detection depends on whether potential cases are recognised at the community level, and whether patients spontaneously present to the HAT treatment centre, or are referred to it by other health facilities. At each of these steps, potential barriers may arise, but published evidence on these barriers is all but missing, with the exception of studies from western Democratic Republic of Congo and neighbouring Republic of Congo. These suggest that local beliefs and illness concepts do play an important role in determining treatment choices, but that biomedical testing and treatment are considered valid ways to decide whether the illness is of biomedical or spiritual origin [3–6].

While not well documented in the literature, structural and financial barriers may also feature prominently in treatment seeking decisions: nearly all gambiense HAT foci occur in areas of great poverty, with minimal transport infrastructure and long distances to negotiate. For other diseases such as malaria and tuberculosis, communities have been shown to make rational decisions that take into account the perceived probability of achieving cure, given the available treatment options, weighed against the costs of seeking care [7–9].

Because of the complex treatment regimens, HAT programmes are not always well integrated with the routine health system. In a study of rhodesiense HAT in eastern Uganda, 77% of patients treated by the HAT centre had not been referred by other health facilities, and 71% had been seen by at least 3 formal health facilities before finally being diagnosed with HAT [10]. Sheer lack of knowledge about the specialised HAT treatment centre’s existence may also explain poor passive screening coverage [11, 12].

The disease progression of gambiense HAT occurs in two stages: The initial haemo-lymphatic phase (stage 1) of the infection is characterised by intermittent, mild and non-specific symptoms such as malaise, headache and fever. Crossing of the blood-brain barrier by the parasite initiates the meningo-encephalitic phase (stage 2) of the disease, which features wasting, organ malfunction, opportunistic infections, and progressively severe neurological symptoms, including personality changes, altered circadian and sleep cycles, and eventually irreversible coma.

The timing of detection is therefore a crucial determinant of the impact of passive case detection: if, for example, all cases were detected passively, but only during the very last phase of stage 2 disease, the effect of case detection would be very substantial in terms of averting HAT deaths, but negligible in terms of reducing stage 2 sequelae (since no progressions from stage 1 would be averted) and prevention of onward transmission.

Indeed, while the clinical impact of passive case detection is obvious, the degree to which it may reduce HAT transmission is unclear. While in general passive case detection may occur late in infection episodes, even a relatively small reduction in the average duration of infectiousness might have a critical effect, especially if the reproduction number is low and thus already close to the extinction threshold. A related question is whether, in practical field conditions, the transmission impact of passive case detection alone is sufficient to create conditions for elimination or at least long lasting control of HAT, without the considerable costs and human resource inputs of active screening. Theoretically, the reduction in transmission could be 100% if all cases were detected immediately after infection.

Past HAT programmes have emphasised maximising the coverage of active screening through community sensitisation and, in the more remote colonial period, coercive measures. By contrast, rarely have attempts been made to study the determinants of low passive screening uptake, and to improve its coverage and timeliness [13].

Here, we generated estimates of the coverage and epidemiological impact of passive case detection for a large number of villages from three Médecins Sans Frontières (MSF) projects in Southern Sudan and Uganda in the late 1990s and early 2000s. At that time, thousands of cases of HAT were reported from these countries, but numbers have fallen dramatically since to 4 reported cases in Uganda and 17 in South Sudan in 2016 [14]. We used a mathematical model of HAT to estimate the transmission intensity and human-to-human reproduction number (*R*_H→H_) of gambiense HAT at the time of the MSF studies. Corollary analyses explored the association of transmission intensity and coverage with certain variables, including the utilisation rate of passive screening.

## Methods

### Ethics statement

The study was approved by the London School of Hygiene and Tropical Medicine Ethics Committee (Approval #3047).

### Data

We identified all instances of consecutive active screenings screening sessions in the Kiri (Southern Sudan, 2000–2007), Adjumani (Uganda, 1991–1996) and Arua-Yumbe (Uganda, 1995–2002) MSF projects, described previously [15–18]. In all projects, cases detected in stage 1 were treated with pentamidine. In the Uganda sites, during the period of this analysis, both standard and short-course first-line regimens of melarsoprol were used for stage 2; relapses received a variety of compassionate single-drug or combination regimens including melarsoprol itself, nifurtimox and eflornithine once the latter became available to MSF.

**Fig 1:**
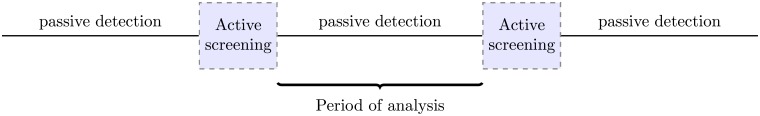
Schematic representation of the data.

We extracted the number of stage 1 and 2 cases detected actively in each of the two screening sessions and passively between them (Fig 1). Time series were only retained if they contained population data, and were from villages with an estimated non-zero HAT prevalence at the time of the first or second active screening session [19], or in which at least one case was detected passively during the inter-screening period.

### Indicators and definitions

We defined he following two passive detection coverage indicators:

- **stage 1 detection coverage**: the number of stage 1 cases detected, out of the total number of period-prevalent stage 1 cases, over a given inter-screening period;
- **stage 2 detection coverage**: the number of stage 2 cases detected, out of the total number of period-prevalent stage 2 cases, over a given inter-screening period.

These indicators express the direct effect of passive case detection on disease progression and death. Similarly, we can quantify incomplete coverage as the

- **risk of stage 1 to 2 progression**: the number of stage 1 to stage 2 progressions, out of the total number of period prevalent stage 1 cases, over a given inter-screening period; and the
- **risk of HAT death**: the number of HAT deaths, out of the total number of period prevalent stage 2 cases, over a given inter-screening period.

### Mathematical model

**Fig 2:**
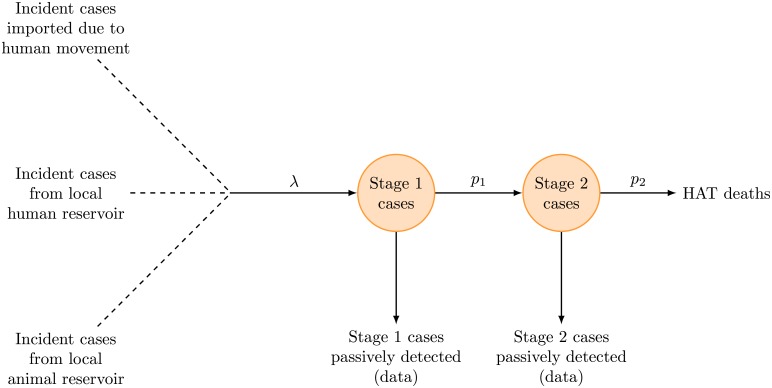
Schematic representation of the model.

We used a stochastic model of HAT transmission with a closed population and constant incidence rate (Fig 2) that varied between villages, implemented with a binomial tau-leap algorithm using a fixed step size of one month. While all HAT infection involves transmission to tsetse flies, we did not explicitly model infection in the fly population to simplify calculations. New stage 1 infections in village *i* were generated among the susceptible population with monthly incidence rate (or monthly probability of a given susceptible person to get infected) λ_*i*_, encompassing transmission due to the human infectious pool within the village, zoonotic infections contracted from a putative animal reservoir, and importation of cases due to travel to other villages or immigration of cases or infected flies. Progression of stage 1 to stage 2 occurred with fixed monthly probability *p*_1_, and progressions from stage 2 to death with monthly probability *p*_2_, both held constant across villages.

By using a constant incidence rate, we assumed that all vector parameters (size of fly population, biting rate, proportion of bites taken on humans, probability that a bite by an infectious fly on a susceptible human results in transmission, probability that a bite by a susceptible fly on an infectious human results in transmission) remained constant during the inter-screening periods, and that all uninfected members of the population were at equal risk of HAT infection. By using closed populations, we assumed that there was no in- and out-migration of cases (i.e., that travel contributed to incidence only through people becoming infected while on a trip away from the village). We further assumed that cases did not experience mortality due to causes other than HAT, as the time series studied were short compared to human lifespans.

The model was implemented in the C++ programming language.

### Accounting for diagnostic accuracy

Diagnosis of HAT usually follows complex algorithms including serological screening followed by parasitological confirmation and staging. Briefly, the algorithm used in the study sites analysed here used an initial Card Agglutination Test for Trypanosomiasis (CATT) [20] for screening and a capillary tube centrifugation (CTC) or the Quantitative Buffy Coat (QBC) technique in Southern Sudan, and the mini anion exchange centrifugation technique (mAECT) or QBC in Uganda for parasitological confirmation [21].

In the model runs for each village, any cases detected passively during each month in the time series were subtracted from the number of current stage 1 and 2 cases, respectively, after correcting for HAT stage misclassification, a known limitation of diagnostic algorithms used at the time. To do this, probabilities $\sigma_{1}^{*}$ and $\sigma_{2}^{*}$ of being correctly classified in stage 1 and stage 2, respectively, were randomly sampled from triangular distributions within previously derived bounds [21]. The true number of stage 1 and 2 cases detected passively, *D*_1_ and *D*_2_, was then randomly sampled from the observed number of stage 1 and stage 2 cases detected *D*_obs,1_ and *D*_obs,2_ using the probabilities

$$P(D_{1},D_{2}|D_{\text{obs},1},D_{\text{obs},2})=\sum_{\begin{array}{c} i=\text{max}(0,D_{\text{obs},2}-D_{2})\\ j=\text{max}(0,D_{\text{obs},1}-D_{1}) \end{array}}^{\begin{array}{c} \text{min}(D_{1},D_{\text{obs},2})\\ \text{min}(D_{2},D_{\text{obs},1}) \end{array}}\sigma_{1}^{*D_{1}-i}{\left(1-\sigma_{1}^{*}\right)}^{i}\sigma_{2}^{*D_{2}-j}{\left(1-\sigma_{2}^{*}\right)}^{j},$$

which are obtained by summing over all possible permutations of *D*_1_ and *D*_2_ that could have led to *D*_obs,1_ and *D*_obs,1_ under the constraints that *D*_1_ + *D*_2_ = *D*_obs,1_ + *D*_obs,2_.

### Estimating the incidence rate

We estimated the incidence rate λ_*i*_, or the probability of each susceptible person to become infected in a given month, in each village *i* using a grid search algorithm. All other parameters were taken from estimates obtained previously using the same data set (Table 1). Initial conditions were derived by subtracting the number of cases of stage 1 and stage 2 detected during the first screening session from the estimated true number of stage 1 and stage 2 cases immediately prior to the screening session [19]. For each village inter-screening time series, we ran the dynamic model from time *t* = 0 until *t* = *T*_*i*_, where *T*_*i*_ is the number of months between the two screening sessions in village *i*, with candidate values of λ_*i*_ ranging from 0 to 0.05 in increments of 0.0001.

**Table 1:** Model parameters. 95% CIs are given in parentheses.

Parameter	Symbol	Values	Source / Notes
Time unit		1 month	
Mean number of days in 1 month	*m*	30.41	
Duration of inter-screening period in village *i*	*T*_*i*_	Variable	Data
Village population size during month *t*	*N*_*t*_	Variable	Data
Probability of correct classification in stage 1	$\sigma_{1}^{*}$	Kiri (old): 0.68 (0.39–0.87)	[21]
		Kiri (new): 0.66 (0.39–0.87)	[21]
		Adjumani: 0.70 (0.39–0.89)	[21]
		Arua-Yumbe: 0.66 (0.39–0.89)	[21]
Probability of correct classification in stage 2	$\sigma_{2}^{*}$	Kiri (old): 0.95 (0.82–0.99)	[21]
		Kiri (new): 0.95 (0.81–0.98)	[21]
		Adjumani: 0.94 (0.79–0.98)	[21]
		Arua-Yumbe: 0.93 (0.79–0.98)	[21]
Daily rate of progression from stage 1 to 2	*r*_1_	0.0019 (0.0012–0.0028)	[22]
Daily rate of death once in stage 2	*r*_2_	0.0040 (0.0025–0.0058)	[22]
Monthly probability of progression from stage 1 to 2	*p*_1_	$1-e^{-mr_{1}}$	from daily rate
Monthly probability of death once in stage 2	*p*_2_	$1-e^{-mr_{1}}$	from daily rate
Monthly incidence rate	λ_*i*_	Variable (0 to 0.05 per person-month)	Estimated

Each candidate value of λ_*i*_ was evaluated with 100 different random numbers of detected cases after correcting for misclassification, in 1,000,000 runs for each of these samples. In every run, random values of *p*_1_ and *p*_2_ were sampled from log-normal distributions with mode and spread given previously derived estimates [22]. The likelihood of each value of λ_*i*_ was evaluated by enumerating the proportion of model runs that yielded the correct (estimated) prevalence of stage 1 and stage 2 cases at the second screening session while maintaining *S*_1_ ≥ 0 and *S*_2_ ≥ 0 at all times during the run.

The best estimate of λ_*i*_ for each village time series was provided by the value with maximum likelihood while confidence intervals (CIs) were obtained from the 95% percentiles of the likelihood profile distribution [23]. Best estimates and 95% CIs of λ across villages in the three project sites were computed through bootstrapping, by (i) drawing 10,000 independent samples with replacement from the likelihood distribution of λ_*i*_ for each village time series; (ii) calculating the weighted mean of each set of sampled values, weights being equal to the total person-time represented by the village time series, i.e. $\sum_{t=0}^{T}N_{t}$; and (iii) computing the median and 95% percentiles of the resulting distribution of 10,000 weighted means.

Once λ was estimated, it was fed back into the model so as to predict the numbers of incident cases, progressions and deaths during each village time series, as well as other interesting statistics. Indicators of the coverage and transmission impact of passive case detection were then obtained from the above numbers. Incidence, coverage and relative transmission impact were also estimated at the project level, through the bootstrapping method described above.

### Estimating detection coverage, transmission potential and the impact of passive detection

To estimate the detection coverage, human-to-human reproduction number *R*_H→H_, and the impact of passive case detection, 1,000,000 iterations of the model were run for each village series; for each run, a random value of λ_*i*_ was sampled with replacement from its likelihood distribution in village *i*.

The impact of passive detection on transmission was calculated as the difference in the cumulative person-time of infectiousness between the actual and counterfactual scenario of zero passive case detection. This corresponds to a reduction in the human-to-human reproduction number *R*_H→H_, which we assumed to be linearly related to the duration of infectiousness. Detection coverage was calculated as the number of stage 1 and 2 cases detected passively, out of the total number of period prevalent stage 1 and 2 cases, respectively. The risk of stage 1 to 2 progression was calculated as the number of stage 1 to stage 2 progressions, out of the total number of period prevalent stage 1 cases, over a given period. The risk of HAT death was calculated as the number of HAT deaths, out of the total number of period prevalent stage 2 cases (excluding detections), over a given period.

Incident cases that occurred in the absence of any prevalent stage 1 or stage 2 cases during the same or the previous month were classified as due to sources of transmission other than the local human infectious pool within the village; all other incident cases were assumed to be due to the human infectious pool. The transmission rate *β*_*i*_ of the infection in village *i*, that is, the number of secondary infections caused by one infectious person per unit time, was calculated as the number of incident cases found to be due to the human infectious pool, per infectious person-month, over the period T:

$$\begin{array}{c} \beta_{i}=\frac{\sum_{t=1}^{T_{i}}S_{1,i,\text{new},\text{human},t}}{\sum_{t=1}^{T_{i}}(S_{1,i,t}+S_{2,i,t})} \end{array}$$

where *S*_1,*i*,new,human,*t*_ is the number of new (incident) cases found to be due to the human infectious pool at time *t* and *S*_*j*,*i*,*t*_ is the number of prevalent cases in village *i* in stage *j* at time *t*. The human-to-human reproduction number is estimated as the transmission rate times the duration of infection, assuming that stage 1 and stage 2 cases are equally transmissible,

$$\begin{array}{c} R_{\text{H}\to \text{H}}=\beta \left(\frac{1}{p_{1}}+\frac{1}{p_{2}}\right) \end{array}$$ (1)

The estimate of *R*_H→H_ should be regarded as an upper limit, since an unknown proportion of incident cases assumed to be due to the human infectious pool in the village may in fact be due to zoonosis or case importation. It refers strictly to the human–fly–human transmission cycle within a given village or project, in the absence of control and assuming full population susceptibility. It is the square of the basic reproduction number *R*_0_ calculated elsewhere with the fly population explicitly modelled [24, 25].

Point estimates and 95% CIs for detection coverage, transmission impact and *R*_H→H_ at the level of each village time series were computed from the bootstrap distributions, by computing the mode and 95% percentile intervals, respectively. Point estimates and 95% CIs at the level of each project were also computed through a bootstrap, with samples weighted by the maximum likelihood estimates of λ_*i*_ as time series with higher incidence contributed more to the denominators of the above indicators.

### Factors associated with the incidence rate and detection coverage

The association between the forces of infection λ_*i*_ and other factors (average village population size during the analysis period, rate of passive case detection during the analysis period, expressed as cases per 1000 person-months) at the level of the village time series was explored in ordinary least squares linear regression models, implemented in the *R* software. A bootstrapping technique was implemented to randomly select 10,000 sets of values of λ_*i*_ from their likelihood profile distributions, and regressions performed on each of these 10,000 sets. Medians and 95% percentile intervals of the coefficients from each of the 10,000 regression outputs are presented.

In order to perform the least squares regression, λ_*i*_ was log transformed to normalise its distribution, while factors were treated as categorical variables. An analytic weight was applied to each observation, equal to *T*_*i*_, and model coefficient standard errors were adjusted for clustering due to repeated observations from the same village.

The association between detection coverage in stage 1 and stage 2 and the passive screening attendance rate during period *T*_*i*_ (expressed as the number of people screened per 1000 person-months) was also explored in a least squares linear regression. For some time series, data on the number of people screened passively were missing for one or more months; for this reason, and in order to account for different amounts of person-time represented by each time series, each observation was weighted by the amount of person-time in the time series for which passive screening attendance data were available.

## Results

### Profile of time series included in the analysis

Overall, 324 village time series were included in the analysis, the majority of which were from the Adjumani project (Table 2). In all three projects, the median duration of the series was around one year. Arua-Yumbe, a comparatively population dense area of northwest Uganda, featured large population units, whereas in Kiri, Sudan most villages were very small (assuming about 6 people per household, a median village size of 207 translates to about 33 households).

**Table 2:** Profile of time series included in the analysis, by HAT project.

	Kiri, Sudan,	Adjumani, Uganda,	Arua-Yumbe, Uganda
Number of time series	70	206	48
Number of time series following a repeat round of active screening (%)	34 (48.6)	126 (61.2)	18 (37.5)
Median duration of the inter-screening period in months (IQR)	11 (8–13)	9 (6–14)	14 (10–24)
Median village population size (IQR)	207 (107–367)	803 (588–1068)	2517 (2083–3129)
Median passive case detection rate in cases per 1000 person-months (IQR)	0.49 (0.00–1.05)	0.30 (0.15–0.70)	0.19 (0.08–0.37)

Overall, about 4.4 million person-months across the three projects were included in the analysis, or 8.1% of about 54.7 million person-months available in the datasets. Most of the remaining person-time was excluded as it comprised periods either before the first or after the last active screening round, or time series from villages in which no active screening or only one active screening ever took place (the majority of villages in all projects were never actively screened or only screened once, and thus did not feature the consecutive screenings required for this analysis).

### Estimates of incidence and detection coverage

Incidence estimates were obtained for 323/324 time series (the model did not yield any solution for one time series from Arua-Yumbe).

**Fig 3:**
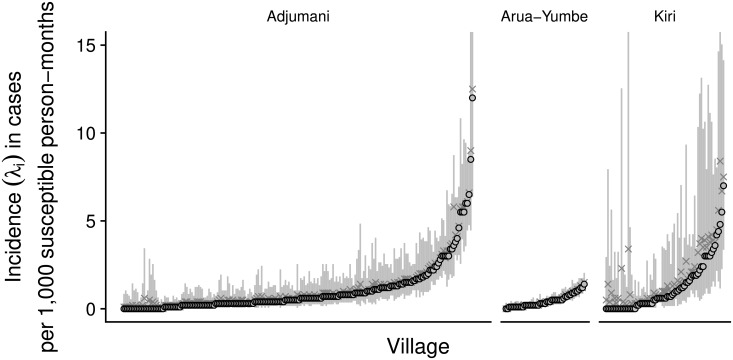
Estimates of the incidence rate, by village time series. Shown are maximum likelihood values (points), median values (crosses) and 95% percentile intervals (vertical lines) for each village time series. Time series are ranked on the x-axis by project and ascending maximum likelihood value. The y-axis is truncated at 15 cases per 1000 susceptible person-months for clarity purposes.

**Fig 4:**
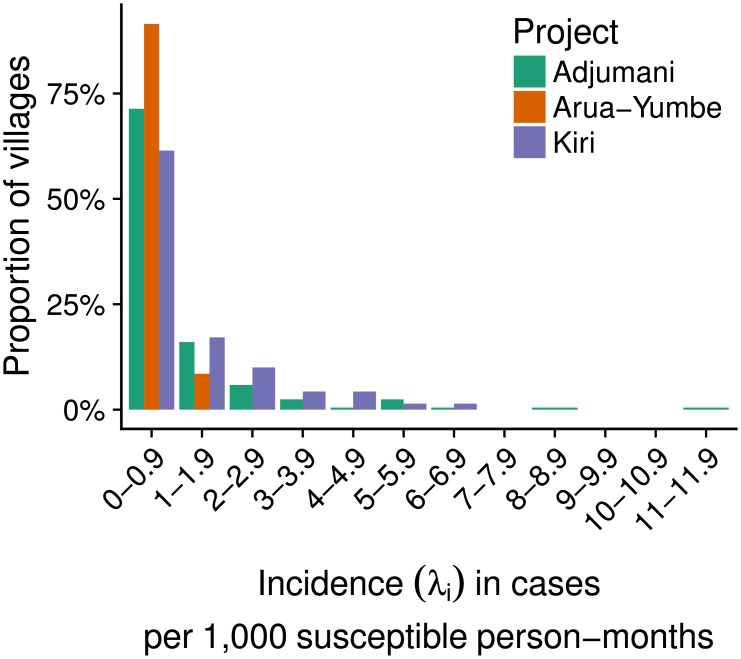
Distribution of maximum likelihood estimates of the incidence rate, by project.

Most estimates were somewhat skewed, and there was variation within each project (Fig 3). Estimates were also fairly imprecise at the village level, particularly in Kiri where the numerators (number of cases) and denominators (village population size) informing the model were, on average, smallest. Nonetheless, the vast majority of maximum likelihood estimates were below 5 cases per 1000 person-months (Fig 4), and high incidence outliers were rare.

For 52/70 (74.3%) of time series in Kiri, 182/206 (88.3%) in Adjumani and 45/47 (95.7%) in Arua-Yumbe, the model predicted non-zero incidence with probability of one. The probability of non-zero incidence was less than 0.5 in only 1/70 (1.4%), 4/206 (1.9%) and 2/47 (4.3%) time series, respectively.

At the project level, estimates of the incidence rate were much more precise (Table 3). They were of similar magnitude in Kiri and Adjumani, while in Arua-Yumbe they were about 50% lower. These rates are between two and three times as high as the respective passive case detection rates during the periods analysed (Table 2).

**Table 3:** Estimates of incidence rate and detection coverage, by HAT project. 95%CIs are given in parentheses.

Parameter	Kiri, Sudan,	Adjumani, Uganda,	Arua-Yumbe, Uganda
Number of incident cases	235 (221–252)	1734 (1674–1799)	836 (806–873)
Incidence rate (cases per 1000 susceptible person-months)	0.92 (0.81–1.04)	0.99 (0.94–1.05)	0.45 (0.42–0.48)
Cases appearing to be not due to human reservoir	44.0% (37.3%–50.7%)	8.2% (6.6%–10.1%)	3.3% (2.0%–5.3%)
Stage 1 detection coverage	29.0% (22.3%–38.4%)	25.6% (23.7%–27.4%)	21.8% (19.5%–24.0%)
Risk of stage 1 to 2 progression	58.1% (49.4%–65.1%)	47.8% (45.7%–49.9%)	50.0% (47.2%–52.6%)
Stage 2 detection coverage	48.6% (38.6%–56.5%)	43.3% (40.1%–46.3%)	63.3% (56.8%–59.1%)
Risk of progression from stage 2 to death	39.6% (31.5%–50.2%)	22.5% (19.4%–25.6%)	28.7% (22.6%–34.7%)

A minimum proportion of 44.0% (95% CI 37.3%–50.7%) of incident cases in Kiri could not be attributed to the human-fly-human cycle within villages. Elsewhere, fewer than 10% of cases met this condition.

We further estimated that, among period-prevalent stage 1 cases, about half progressed to stage 2 without being detected passively. Stage 1 detection coverage was less than 30% in the three projects. Among stage 2 cases a greater proportion were detected, but a sizable minority progressed to death without being detected during the period. When divided by the total person-time at risk in the time series, the predicted HAT deaths amounted to a mean estimated HAT specific mortality rate of 3.8, 5.4 and 2.3 per 1000 person-years in Kiri, Adjumani and Arua-Yumbe, respectively.

### Factors associated with incidence and detection coverage

There was some indication (*p* for trend < 0.05) of decreasing incidence rate with increasing population size (Table 4). There was also evidence of a positive association (*p* for trend < 0.001) between incidence and passive case detection rate.

**Table 4:** Association of incidence rate with village population size and passive case detection rate. *Coefficient* is the median adjusted coefficient, and *p-value* the median adjusted p-value, both with 95% confidence intervals given in parentheses.

Factor	Coefficient	p-value
**Village population size**		
<250	reference	
250–499	-1.08 (-2.05–(-0.08))	0.074 (0.001–0.776)
500–999	-1.52 (-2.42–(-0.53))	0.017 (<0.001–0.425)
1000–1999	-1.66 (-2.55–(-0.69))	0.013 (<0.001–0.314)
≥ 2000	-2.31 (-3.17–(-1.24))	0.015 (<0.001–0.182)
**Passive case detection rate** (per 1000 person-months)		
0	reference	
0.01–0.49	2.51 (1.60–3.39)	<0.001 (<0.001–0.005)
0.50–0.99	3.39 (2.51–4.31)	<0.001 (<0.001–<0.001)
1.00–1.99	3.91 (3.04–4.83)	<0.001 (<0.001–<0.001)
≥ 2.00	4.27 (3.40–5.25)	<0.001 (<0.001–<0.001)
**Project**		
Adjumani	reference	
Arua-Yumbe	0.11 (-0.29–0.37)	0.746 (0.378–0.990)
Kiri	-0.42 (-1.05–0.10)	0.381 (0.051–0.944)

### Reproduction numbers and impact of passive case detection on transmission

Considering only incident infections that could be attributed to the human-fly-human cycle within villages, mean estimates of the upper limit of the human-to-human reproduction number *R*_H→H_ ranged from 3.1 to 5.7 (Table 5). Passive case detection was estimated to have reduced *R*_H→H_ by 31.6–48.4% compared to a scenario of no passive case detection.

**Table 5:** Estimates of the human-to-human reproduction number and transmission impact of passive detection, by HAT project. 95%CIs are given in parentheses.

Parameter	Kiri, Sudan,	Adjumani, Uganda,	Arua-Yumbe, Uganda
Upper limit of *R*_H→H_	3.1 (2.2–4.7)	5.7 (4.5–8.1)	4.0 (3.1–5.6)
Impact of passive detection	43.1% (38.2%–48.4%)	39.9% (37.2%–42.4%)	33.1% (31.6%–34.6%)

## Discussion

### Interpretation of findings

Passive case detection has received relatively little attention in the study of HAT epidemiology, yet it is a means whereby much surveillance and case detection take place, and it also provides much of the information on incidence used to quantify the burden of HAT locally and globally.

This study provides what appear to be the first estimates of the coverage of stage 1 and 2 passive case detection in gambiense HAT control programmes. Findings suggest that only a minority of stage 1 cases are detected by this method of control, i.e. that in a situation without active screening most pathogenic infections would progress to stage 2. These observations are broadly consistent with those reported from a variety of other HAT foci [26–33]. Once in stage 2, about half of cases were detected during the course of the inter-screening period, and a good number among those remaining were detected via active screening.

Though based on an entirely different estimation approach, these coverage estimates are broadly similar to those obtained for *T. brucei rhodesiense* epidemics in Tororo, Uganda (20% for stage 1 and 42% for stage 2) [34] and nearby Serere (about 60%) [35]. It must be noted, however, that stark differences exist in surveillance, disease progression and treatment between gambiense and rhodesiense HAT [36]. Whether observed similarities are a coincidence or the result of similar underlying systemic and behavioural factors therefore remains an open question.

We also estimated the true incidence of HAT in a large number of village time series. These estimates represent a very considerable disease burden: assuming a mean duration of infection of 26 months [22], and using the simple rule of (prevalence = incidence x duration) whilst assuming stable incidence in a closed population, these estimates would translate, in the villages included in our dataset, into a prevalence of about 2.5% in Adjumani and Kiri, and 1.2% in Arua-Yumbe. They also imply that, in the absence of case detection and assuming all infections are pathogenic, about 0.5 to 1% of the total population would get infected with HAT and thus die, per year: by comparison, a typical crude death rate in Sub-Saharan Africa is 1.5 to 2% per year, i.e. HAT in these communities would result in a 30 to 60% increase in all cause mortality. However, because of case detection and the fact that the predictions were censored at the end of the time series, predicted HAT-specific mortality rates were in fact lower.

Considerable heterogeneity in incidence was obvious, with a small minority of villages found to have much higher transmission rates and burdens. As was previously found with active case detection [19], larger population sizes were associated with lower burden in relative terms: possible explanations include: (i) a higher fly-to-human ratio in the smaller villages; and (ii) greater exposure to flies in smaller villages, particularly if village size is a proxy of proximity to vector habitats or greater occupational risk. Conversely, as expected passively observed incidence was strongly associated with true incidence: therefore, prioritising villages for control according to observed caseload seems like an appropriate strategy.

This study raises questions about the possible contribution of animal reservoirs to transmission [24]. *T. b.gambiense* are known to perpetuate, asymptomatically, in both human and non-human warm-blooded animals which share ecosystems with tsetse flies [37]. While we had no other data to validate our method to distinguish likely infections due to non-human-fly-human cycle dynamics, it does suggest marked differences between Kiri (44%) and the Ugandan foci (3–8%). The Sudanese focus has been suggested as the source of epidemics in neighbouring Moyo, Uganda and Nimule, Sudan [38]: this study raises the hypothesis that an animal reservoir might contribute to its persistence. More generally, there is also an urgent need to identify diagnostic markers of the parasite in both humans and animals, as sustained elimination could be threatened by cryptic reservoirs.

The human-to-human reproduction numbers here are large compared to the ones estimated in other settings [24, 25]. Correspondingly, the reductions in transmission achieved through passive case detection are not sufficient to interrupt transmission alone, and would have to increase considerably to do so. In this context, it should be stressed that all estimates arising from this study apply only to inter-screening periods. They are unlikely to be representative of conditions before the first active screening or after the last active screening in a given village. Indeed, the time series included in this analysis feature generally high incidence, and are thus representative of the upper end of the possible range of transmission rates and reproduction numbers. On the other hand, these villages contribute a disproportionate amount of cases, and are critical from the standpoint of control, as they probably fuelled the spatial dynamics of transmission throughout the focus.

### Study limitations

The main limitations of this study are closely tied to some of the modelling assumptions.

The estimates of coverage imply the assumption that, once detected, all cases were treated and recovered. In reality, about 5-10% across the MSF projects died or defaulted treatment [39]. The coverage would therefore have to be slightly adjusted downward to arrive at the true impact on mortality or stage 2 sequelae.

A necessary assumption, in view of the absence of any vector data, was that all vector parameters (e.g. density, death rate) and vector-host interactions (e.g., degree of anthropophily) remained constant throughout the time series. This assumption was almost certainly not met in reality, though to an unknown degree. Similarly, the estimates of the incidence rate should be viewed as averages for the period to which they refer. Bias would have resulted if a secular trend had been ongoing throughout the HAT focus: for example, if vector density had been declining progressively, high incidence at the beginning of the period and low incidence at the end would have resulted in a proportionately higher period prevalence of stage 1 cases than predicted by the model due to early accumulation of stage 1 cases, and thus an overestimation of stage 1 detection coverage.

Our estimates are also sensitive to the assumption that stage 1 and stage 2 cases are equally infectious. A decline in infectiousness in stage 2 might considerably reduce the transmission effect of passive case detection. While very ill stage 2 cases may be bedridden and thus inaccessible to flies, clinical experience suggests that this only encompasses a brief (and therefore epidemiologically negligible) period of days/weeks before death or care-seeking. There is likewise insufficient evidence on infectiousness per fly bite by stage: stage 2 cases might have increased parasitaemia, but parasites might also be mostly sequestered away from peripheral blood vessels.

The results of this study are dependent on the validity of previous work, including estimation of stage 1 and 2 progression rates [22] and staging inaccuracy [21]. Much sensitivity analysis was already built into the model, by allowing probabilistic distributions of most of the above parameters. The starting and ending conditions of the time series, i.e. starting and ending prevalence, are a particularly crucial input: had these been underestimated before [19], detection coverage would have been overestimated here, as a higher starting and ending prevalence implies greater incidence rate during the period, i.e. a larger denominator of period-prevalent cases based on which to calculate coverage.

Lastly, care must be taken in extrapolating these results to other settings. In particular, passive detection coverage is a function of health system capacity, and this may differ significantly in other places.

### Conclusions

Our findings suggest that, even in well-established projects enjoying stable funding and considerable resources (by no means the rule across HAT control programmes today), the reported burden of gambiense HAT is subject to considerable underestimation. Furthermore, despite several limitations this study demonstrates the importance and difficulty of developing methods to measure the impact of control programmes against neglected tropical diseases, for many of which detection coverage is a key determinant.

For gambiense HAT specifically, this study suggests that there is great potential impact in improving passive case detection, which has the potential to detect a large proportion of cases, at a fraction of the cost of active screening. The deployment of oral drugs and rapid diagnostic tests, now a realistic prospect, offers an opportunity to considerably increase passive detection rates and shift the average timing of detection towards stage 1, by decentralising diagnosis and treatment to outpatient health facilities, where stage 1 cases with non-specific symptoms would likely initially present [40, 41]. In this context, mapping fixed health facilities in at-risk areas could play a crucial role in identifying gaps in passive detection coverage [42].

Alternatively, relatively cheap modalities of case detection that combine aspects of active and passive screening, involving existing networks of community health workers to test suspect cases, could be employed. More generally, any approach towards greater emphasis on passive case detection will need to ensure any existing capacity for passive surveillance (e.g., training and knowledge of physicians) is maintained even if active surveillance is scaled down. Passive surveillance requires a strong enough health system with sufficient attendance rates to allow the multiple visits to health facilities that are required for correct diagnosis and treatment. Improving passive surveillance will require engagement with the affected communities and a willingness to tackle structural barriers, such as lack of transport or access to health services, that all too often preclude access to diagnosis and, consequently, treatment for one of the deadliest diseases known to man. As the disease is being pushed towards elimination [25, 43, 44], passive detection will be an ever-more important part of control strategies of gambiense HAT, and estimates of its coverage and efficacy should be crucial components of post-elimination strategies.

## Supporting information

S1 Data Data supplement. The data set used in this study.
(CSV)
